# Dendritic and axonal targeting patterns of a genetically-specified class of retinal ganglion cells that participate in image-forming circuits

**DOI:** 10.1186/1749-8104-9-2

**Published:** 2014-02-05

**Authors:** Jason W Triplett, Wei Wei, Cristina Gonzalez, Neal T Sweeney, Andrew D Huberman, Marla B Feller, David A Feldheim

**Affiliations:** 1 Molecular, Cell and Developmental Biology, University of California, Santa Cruz, 1156 High Street, Santa Cruz, CA 95064, USA; 2 Center for Neuroscience Research, Children’s National Medical Center, 111 Michigan Ave, Northwest, Washington, DC 20010, USA; 3 Molecular and Cell Biology and Helen Wills Neuroscience Institute, University of California, Berkeley, CA 94720, USA; 4 Neurobiology, University of Chicago, Chicago, IL 60637, USA; 5 Neurosciences Department, School of Medicine, University of California, La Jolla, San Diego, CA 92093, USA; 6 Neurobiology Section, Division of Biological Sciences, University of California, La Jolla, San Diego, CA 92093, USA

## Abstract

**Background:**

There are numerous functional types of retinal ganglion cells (RGCs), each participating in circuits that encode a specific aspect of the visual scene. This functional specificity is derived from distinct RGC morphologies and selective synapse formation with other retinal cell types; yet, how these properties are established during development remains unclear. Islet2 (Isl2) is a LIM-homeodomain transcription factor expressed in the developing retina, including approximately 40% of all RGCs, and has previously been implicated in the subtype specification of spinal motor neurons. Based on this, we hypothesized that Isl2^+^ RGCs represent a related subset that share a common function.

**Results:**

We morphologically and molecularly characterized Isl2^+^ RGCs using a transgenic mouse line that expresses GFP in the cell bodies, dendrites and axons of Isl2^+^ cells (Isl2-GFP). Isl2-GFP RGCs have distinct morphologies and dendritic stratification patterns within the inner plexiform layer and project to selective visual nuclei. Targeted filling of individual cells reveals that the majority of Isl2-GFP RGCs have dendrites that are monostratified in layer S3 of the IPL, suggesting they are not ON-OFF direction-selective ganglion cells. Molecular analysis shows that most alpha-RGCs, indicated by expression of SMI-32, are also Isl2-GFP RGCs. Isl2-GFP RGCs project to most retino-recipient nuclei during early development, but specifically innervate the dorsal lateral geniculate nucleus and superior colliculus (SC) at eye opening. Finally, we show that the segregation of Isl2^+^ and Isl2^-^ RGC axons in the SC leads to the segregation of functional RGC types.

**Conclusions:**

Taken together, these data suggest that Isl2^+^ RGCs comprise a distinct class and support a role for Isl2 as an important component of a transcription factor code specifying functional visual circuits. Furthermore, this study describes a novel genetically-labeled mouse line that will be a valuable resource in future investigations of the molecular mechanisms of visual circuit formation.

## Background

The retina performs a wide range of visual processing, including motion detection, color discrimination, and adaptation to changes in light level. This processing is accomplished by parallel circuits in the retina that are comprised of connections between specific types of the six retinal neuronal classes. At the output of each circuit is a unique type of retinal ganglion cell (RGC). RGCs can be classified into approximately 20 subtypes based on molecular, morphological and functional distinctions [1]. How this RGC diversity is established remains unclear, and both activity-dependent [2,3] and -independent [4-6] mechanisms have been proposed. Much of RGC type-specific morphology and functionality is established before eye opening and genetic mechanisms likely play an instructive role in RGC specification. Indeed, cell type specification in a number of systems is driven by regulated expression of transcription factors [7-9], including the differentiation of RGCs [10,11]. However, the factors important for RGC subtype specification remain unclear.

RGCs target several retinorecipient nuclei, including the dorsal lateral geniculate nucleus (dLGN) of the thalamus and the superior colliculus (SC), which are organized topographically. Thus, each region of the dLGN and SC receives input from multiple RGC types, relaying the wide range of visual inputs and contributing to post-synaptic receptive field properties. Receptive field properties of neurons in the dLGN and SC are different from those of RGCs [12,13], and understanding how this visual processing is achieved is dependent on determining which RGC subtypes contribute to the receptive field properties of post-synaptic cells [14].

Islet2 (Isl2) is a LIM homeodomain-containing transcription factor that plays a critical role in the development and differentiation of visceral motor neurons in the spinal cord [15]. Isl2 is also expressed in the retina, beginning at embryonic day 13.5 (E13.5), in post-mitotic cells of the inner and outer retina [16]. As development proceeds, Isl2 expression becomes restricted to the ganglion cell layer (GCL), where it is expressed in approximately 40% of all RGCs. Previous studies show that Isl2 plays a critical role in determining the laterality of RGC projections arising from the ventral-temporal retina [16], but its role in fate specification in the retina remains unclear. Based on this expression pattern in the retina and previously described functions, Isl2 is ideally situated to mediate RGC cell type specification.

Here, we use a novel mouse line that expresses green fluorescent protein (GFP) in the cell soma, dendrites and axons of Isl2^+^ RGCs to determine their morphological and molecular identity. We found that a majority of alpha-RGCs, labeled by the phosphoprotein SMI-32, are GFP^+^ in these mice. Morphological characterization of single cells revealed that most GFP^+^ RGCs are monostratified in sublayer S3 of the inner plexiform layer (IPL), with axons that primarily innervate the dLGN and SC. Finally, previously-described direction selective retinal ganglion cells (DSGCs) and non-DSGCs are shown to be Isl2^-^ and Isl2^+^, respectively, and each can be segregated from one another in the SC by ectopic expression of EphA3 in Isl2^+^ RGCs, providing an important tool for determining the contribution of each RGC type to visual processing.

## Results

### Isl2-GFP BAC transgenic mice label the cell soma, dendrites and axons of a subset of retinal cells

**Figure 1:**
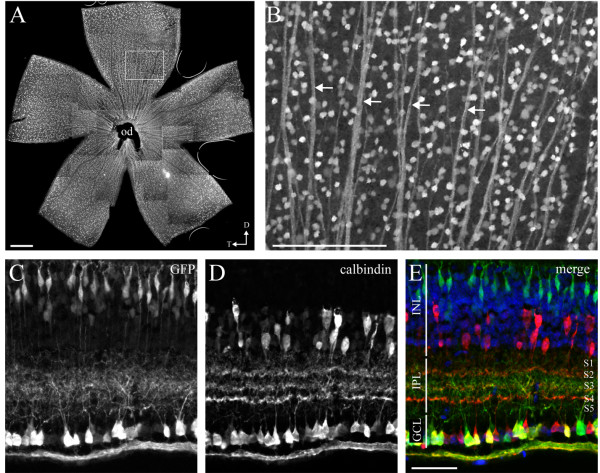
**Subsets of retinal cells are labeled in Isl2-GFP mice. (A)** Immunofluorescent staining of GFP in a retinal flat mount preparation from an adult (P30) Isl2-GFP mouse. od, optic disc; D, dorsal; T, temporal; bar, 200 μm; (**B)** Higher magnification of the image in **(A)** reveals GFP expression in multiple cell bodies and axons (arrows), indicating labeling of RGCs. bar, 200 μm; **(C-E)** Cross section through an adult Isl2-GFP retina reveals GFP expression in cells of the inner nuclear layer and RGC layer; **(C)** Co-staining against the amacrine/RGC marker calbindin reveals a restriction of Isl2-GFP^+^ arbors to S3 of the inner plexiform layer **(E)**. bar, 50 μm.

In order to determine which RGC types express Isl2 during development, we obtained Isl2-GFP bacterial artificial chromosome (BAC) transgenic mice from the GENSAT project [17]. These animals are viable and fertile, and expression of GFP is primarily restricted to the retina, although expression was also detected in the hippocampus, brainstem, and spinal cord (data not shown). As visualized in a retinal flat mount preparation, GFP is expressed in many cells scattered across the retina (Figure 1A). This pattern of distribution is consistent with previous reports of Isl2 expression using *Islet2-LacZ* knock-in mice, suggesting that GFP expression is a relatively reliable replication of endogenous Isl2 expression [16]. Closer examination of GFP expression visualized in retinal flat mount preparations revealed clearly labeled bundles of axons, suggesting that at least a subset of GFP^+^ cells in these mice are RGCs (Figure 1B, arrows). Cross-sectional analysis showed that in addition to cells in the GCL, GFP is also expressed in cells in the inner nuclear layer (INL), which resemble bipolar cells. Interestingly, GFP^+^ arbors within the IPL are predominantly restricted to only the central sublaminae. Indeed, co-labeling with the amacrine/RGC marker calbindin showed that Isl2-GFP^+^ arbors laminate primarily in layer S3 of the IPL, in between the inner and outer calbindin-labeled bands (Figure 1E).

### Isl2-GFP expression closely replicates endogenous Isl2 expression in the GCL

**Figure 2:**
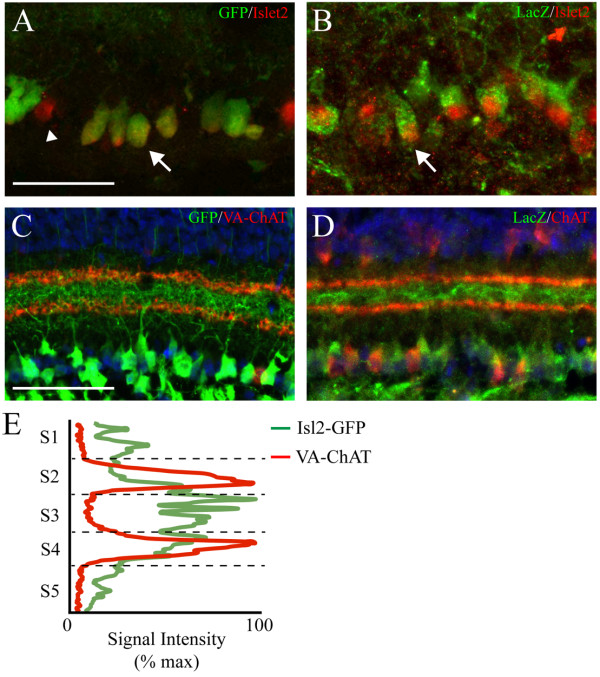
**Isl2-GFP**^**+ **^**expression mimics endogenous Isl2**^**+ **^**expression. (A and B)** Immunofluorescent staining for Isl2 expression (red) in the GCL of P8 Isl2-GFP mice (**A**, green) and Isl2-LacZ knock-in mice (**B**, green) reveals a high degree of co-localization (arrows), although a few Isl2^+^ cells are not GFP^+^ (arrowhead). bar, 50 μm. **(C and D)** Immunofluorescent staining for vesicle-associated choline acetyl transferase (VA-ChAT) (**C**, red) or ChAT (**D**, red) in P15 Isl2-GFP **(C)** and Isl2-LacZ knock-in **(D)** retinas reveals a similar pattern of dendritic arborization in the inner plexiform layer. bar, 50 μm. **(E)** Quantification of fluorescence intensity signal for VA-ChAT (red) and GFP through the IPL of a representative Isl2-GFP retina.

To determine how faithfully GFP expression marks cells that endogenously express Isl2, we determined the extent to which GFP expression and immunoreactivity to an Isl2 antibody overlapped (Figure 2A). As a control, we also determined the extent of overlap of Isl2 anitbody signal with that of β-galactosidase (LacZ) in *Islet2-LacZ* knock-in retinas (Figure 2B), which express LacZ from the Isl2 locus [15]. At postnatal day 8 (P8), Isl2 protein was detected in high proportions of both Isl2-GFP^+^ (89.3%, 50/56 GFP^+^ cells) and Isl2-LacZ^+^ (96.8%, 61/63 LacZ^+^ cells) cells, demonstrating that Isl2-GFP is expressed in most endogenously-expressing Isl2^+^ RGCs. In further support of this conclusion, the dendritic arborizations of Isl2-GFP and Isl2-LacZ expressing retinal cells are strikingly similar (Figure 2C and D). Immunostaining for the starburst amacrine cell markers, choline acetlytransferase (ChAT) or vesicle-associated ChAT (VA-ChAT), labels layers S2 and S4 of the IPL. Co-labeling for GFP in Isl2-GFP retinas reveals a dense stratification of arbors between the two ChAT bands, which was also observed when we co-stained for LacZ in *Islet2-LacZ* retinas (Figure 2C and D). Taken together, these data suggest that GFP expression in RGC layer of Isl2-GFP retinas largely overlaps with that of endogenous Isl2.

### Isl2-GFP is expressed in ON-cone bipolar cells, rod bipolar cells, amacrine cells, and RGCs

**Figure 3:**
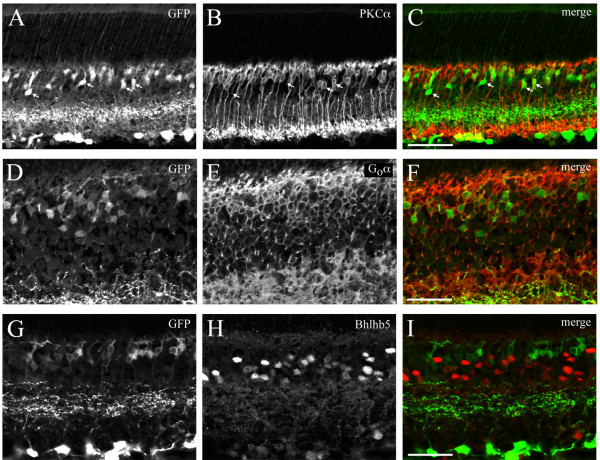
**On-bipolar cells are labeled in Isl2-GFP mice. (A-C)** Immunofluorescent staining of the rod bipolar marker PKCα reveals a high degree of co-localization in Isl2-GFP^+^ cells (arrows). bar, 50 μm; **(D-F)** Immunofluorescent staining for the On-bipolar marker G_O_α reveals co-expression in all GFP^+^ cells in the INL. bar, 50 μm; **(G-I)** Immunofluorescent staining for the Off-bipolar marker Bhlhb5 reveals no co-localization with GFP^+^ cells in the inner nuclear layer. bar, 50 μm.

To begin to characterize the subtypes of retinal cells that are marked in Isl2-GFP retinas, we used immunohistochemical staining of previously established molecular markers of different cell types. First, in the INL, GFP^+^ cells have bipolar cell morphology, and express known markers of both rod and ON-cone bipolar cells. We found that a majority of GFP^+^ cells in the INL were rod bipolar cells (61.2%, 41/67 GFP^+^ cells), indicated by positive co-staining with protein kinase C-alpha (PKCα) (Figure 3A-C) [18,19]. Consistent with this, all GFP^+^ cells examined in the INL were positive for the small G-protein, G_o-_α, which marks ON-bipolar cells of both the rod and cone subtype [20]. Further, staining with the OFF-bipolar marker, Bhlhb5 [21], revealed no co-localization (0/20 GFP^+^ cells), indicating that Isl2-GFP mice do not express GFP in OFF-bipolar cells.

**Figure 4:**
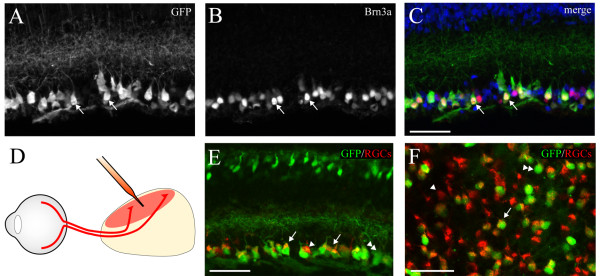
**Retinal ganglion cells (RGCs) are marked in Isl2-GFP mice. (A-C)** Immunofluorescent staining of the RGC marker Brn3a reveals co-localization (arrows) in a subset of Isl2-GFP^+^ cells in a P15 retina. bar, 50 μm. **(D)** Schematic of retrograde tracing strategy to specifically label RGCs. **(E and F)** Fluorescent micrographs of retrogradely labeled RGCs (red) in Isl2-GFP mice in cross-section **(E)** and flat mount **(F)** reveals subsets of co-labeled cells (arrows), retrogradely-labeled-only cells (arrowheads) and GFP-only cells (double arrowheads). bar, 50 μm.

Next, we co-stained Isl2-GFP retinas for Brn3a, a transcription factor important in RGC subtype specification and expressed in approximately 80% of developing RGCs [11,22], to determine the relative subtype composition of GFP^+^ cells in the GCL. A significant portion of Isl2-GFP^+^ cells co-expressed Brn3a at P15 (94.3%, 66/70 GFP + cells) (Figure 4A-C), indicating that most Isl2-GFP^+^ cells in the GCL are RGCs. To complement this molecular labeling strategy, we used a second method to determine if Isl2^+^ cells in the GCL are RGCs. We retrogradely labeled RGCs projecting to the SC, a major target of Isl2-GFP^+^ RGCs, and determined the amount of overlap between retrogradely-labeled cells and Isl2-GFP^+^ cells in the GCL (Figure 4). Using this strategy, we found that 76.3% (495/648, n = 4 retinas) of GFP^+^ cells in the GCL were co-stained with the retrograde label (Figure 4E and F), and 39.6% (495/1250) of the retrogradely-labeled RGCs were also GFP^+^, consistent with previous studies reporting that Isl2-LacZ^+^ RGCs represent approximately 40% of the total RGC population [23]. Because retrograde labeling from the SC does not label all RGCs, it is difficult to determine if the remaining unlabeled GFP^+^ cells are RGCs or displaced amacrine cells. However, Isl2^+^ RGCs target the dLGN and SC primarily and previous studies suggest that the majority of RGCs that project to the SC also project to the dLGN and few RGCs project solely to the dLGN [23,24]. Taken together, these data suggest that Isl2-GFP expression marks a subset of bipolar cells, amacrine cells and RGCs in the retina.

### Isl2-GFP RGCs have common molecular and morphological properties

**Figure 5:**
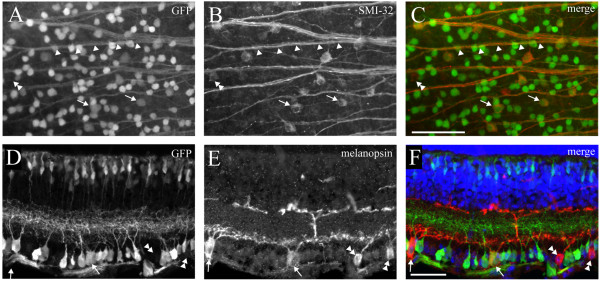
**The majority of putative alpha retinal ganglion cells (RGCs) are Isl2-GFP**^**+**^**. (A-C)** Immunofluorescent staining of the putative alpha cell marker, SMI-32 **(B)**, and GFP **(A)** in a flat mount Isl2-GFP retina reveals a high degree of co-localization in both cell bodies (arrows) and axons (arrowheads). bar, 100 μm. **(D-F)** Immunofluorescent staining of the intrinsically photosensitive RGC marker, melanopsin **(E)**, and GFP **(D)** in an Isl2-GFP retina reveals a subset of co-labeled cells (arrows) and single-labeled cells (double arrowheads). bar, 50 μm.

We next wanted to determine which subtypes of RGCs are represented in the Isl2-GFP population. Strikingly, we found a clear co-localization of Isl2-GFP with the putative alpha-RGC marker, SMI-32 (Figure 5A-C), with 82.9% (63/76) of SMI-32^+^ cell somas and axon bundles also expressing GFP (Figure 5A-C, arrowheads). We also found that a small proportion of GFP^+^ RGCs express melanopsin, a marker of intrinsically photosensitive RGCs (ipRGCs), with 3.6% (4/112) of GFP^+^ RGCs co-expressing melanopsin (Figure 5D-F). Based on previous studies, we hypothesize that Isl2-GFP^+^ ipRGCs may be previously described M4 ipRGCs [25,26], which are related to ON-alpha cells described morphologically [27].

**Figure 6:**
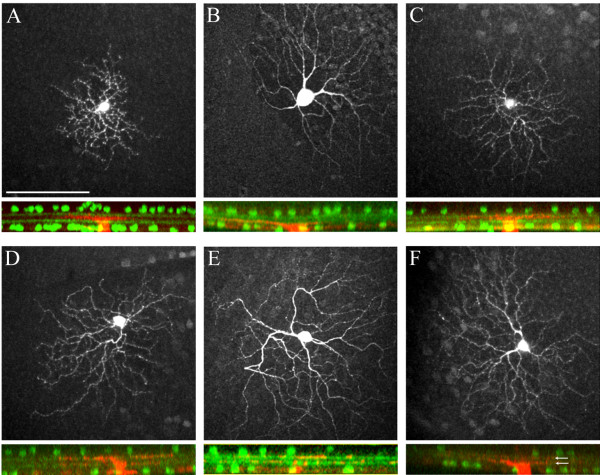
**Isl2-GFP**^**+ **^**retinal ganglion cells (RGCs) have common morphological features. (A-C)** (top) Fluorescent micrographs of Isl2-GFP^+^ RGCs filled with biocytin to visualize their dendritic arborizations in whole mount. bar, 100 μm. **(A-C)** (bottom) Two-photon z-stack through inner retinas in which Isl2-GFP^+^ RGCs were filled with biocytin (red) and ChAT was detected by immunofluorescence (green). **(D-F)** (top) Fluorescent micrographs of Isl2-GFP^-^ RGCs in an Isl2-GFP^+^ retina filled with biocytin to visualize their dendritic arborizations in whole mount. **(D-F)** (bottom) Two-photon z-stack through inner retinas of Isl2-GFP mice in which Isl2-GFP^-^ RGCs were filled with biocytin (red) and ChAT was detected by immunofluorescence (green, arrows in F).

To further characterize the dendritic architecture of Isl2-GFP RGCs, we filled individual GFP^+^ and GFP^-^ cells in Isl2-GFP retinas with biocytin. We found that while both the cell body and dendritic field size were variable among Isl2-GFP^+^ RGCs (Figure 6A-C), they did share a number of characteristics in their dendritic lamination patterns. First, we found that 83.3% of Isl2-GFP^+^ RGCs (15/18 cells) have dendrites that are located in between the ChAT laminae (11 monostratified and 4 bistratified) and the remaining 3 RGCs have dendrites above and below the ChAT laminae. Second, we found that none of the dendrites of Isl2-GFP^+^ RGCs co-localized with the ChAT laminae. Finally, we found that 40% (4/10) of Isl2-GFP^-^ cells had dendrites that were bistratified and co-localized with ChAT bands in the IPL (Figure 6D-F). Taken together, these molecular and morphological data suggest that the majority of Isl2-GFP^+^ cells in the GCL are RGCs that are comprised of multiple previously-described subtypes, but few of these are ON-OFF DSGCs.

### Isl2-GFP RGCs project to the dLGN and SC

**Figure 7:**
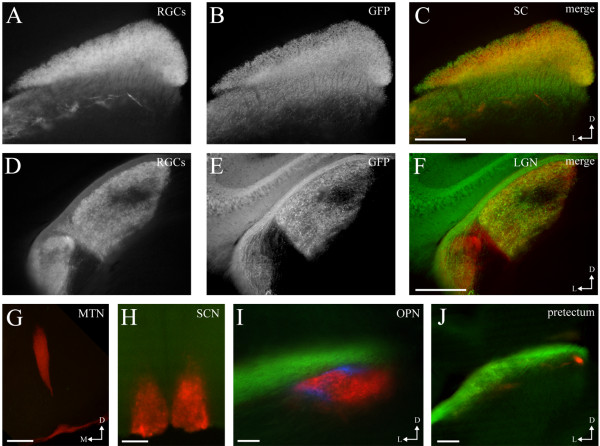
**Isl2-GFP**^**+ **^**retinal ganglion cells (RGCs) project to the superior colliculus (SC) and lateral geniculate nucleus (LGN). (A-C)** Coronal sections through the SC of an Isl2-GFP mouse in which all RGCs were labeled with fluorescently-conjugated cholera toxin subunit B (CTB-555) (red) reveals dense innervation by Isl2^+^ RGCs (green). D, dorsal; L, lateral; bar, 250 μm. **(D-F)** Coronal sections through the lateral geniculate nucleus (LGN) of an Isl2-GFP mouse in which all RGCs were labeled with CTB-555 reveals innervation in the contralateral zone of the dorsal LGN and avoidance of the ventral LGN and intergeniculate leaflet. D, dorsal; L, lateral; bar, 250 μm. **(G-J)** Coronal sections through the medial tegmental nucleus (MTN) **(G)**, suprachiasmatic nucleus (SCN), olivary pretectal nucleus (OPN) **(I)** and the pretectum **(J)** of an Isl2-GFP mouse in which all RGCs were labeled with CTB-555 reveals a lack of innervation of these areas by Isl2-GFP^+^ RGCs. D, dorsal; M, medial; L, lateral; bar, 100 μm.

RGCs relay visual information to several targets in the brain that differ in their function. To determine if Isl2^+^ RGCs participate in specific visual circuits, we determined which visual nuclei are innervated by Isl2-GFP^+^ RGCs by labeling all RGCs with fluorescently-tagged cholera toxin subunit B (CTB-555) to identify all retinorecipient areas. We found that in adult mice, Isl2-GFP^+^ axons primarily innervate the SC and dLGN of the thalamus, with a few fibers terminating in the ventral LGN (vLGN) (Figure 7A-F). Importantly, Isl2-GFP^+^ RGCs do not terminate in areas thought to be important for non-image forming visual functions, such as the suprachiasmatic nucleus (SCN), olivary pretectal nucleus (OPN), pretectal complex (PTC) or medial tegmental nucleus (MTN) (Figure 7G-J). Interestingly, Isl2-GFP^+^ RGCs project only to the contralateral domains of the SC and dLGN, avoiding the ipsilateral patches in each nucleus. This is consistent with previous studies showing that Isl2^+^ RGCs project only contralaterally in the dLGN [16]. In each of these areas, Isl2-GFP^+^ RGCs terminate in all sublayers, again suggesting that multiple subtypes of RGCs express Isl2 [28,29].

**Figure 8:**
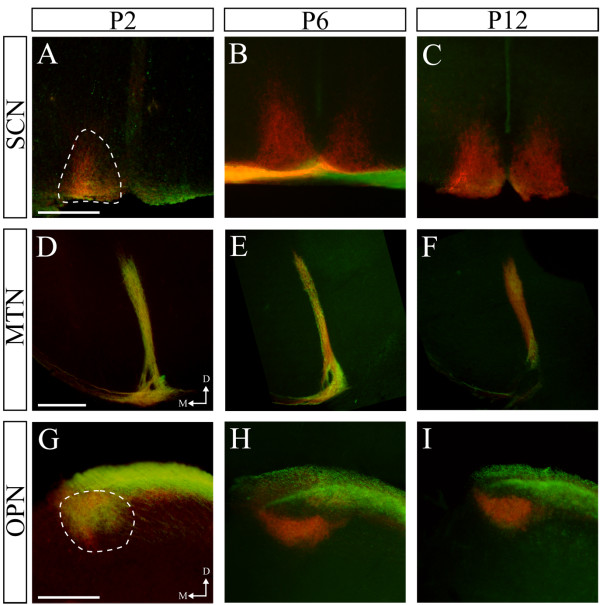
**Isl2-GFP**^**+ **^**retinal ganglion cells (RGCs) project to the medial tegmental nucleus (MTN) and olivary pretectal nucleus (OPN) early in development. (A-C)** Coronal sections through the SCN of P2 **(A)**, P6 **(B)** and P12 **(C)** Isl2-GFP mice in which all RGCs were labeled with CTB-555 (red) reveal a lack of innervation at any point by Isl2-GFP^+^ RGCs (green). **(D-F)** Coronal sections thought the MTN of P2 **(D)**, P6 **(E)** and P12 **(F)** Isl2-GFP mice in which all RGCs were labeled with CTB-555 reveals an early innervation by Isl2-GFP^+^ RGCs. **(G-I)** Coronal sections through the OPN of P2 **(G)**, P6 **(H)** and P12 **(I)** Isl2-GFP mice in which all RGCs were labeled with CTB-555 reveal an early innervation by Isl2-GFP^+^ RGCs which is quickly pruned by P6. D, dorsal; M, medial; bar, 200 μm.

We next asked if Isl2-GFP^+^ RGCs change their projection patterns during development by determining the projection patterns of Isl2-GFP^+^ axons at early postnatal ages. We found that Isl2-GFP^+^ RGCs innervate the SCN, MTN and OPN at early postnatal ages in contrast to their pattern of innervation at later ages (Figure 8). Whether these arbors are selectively pruned, if Isl2^+^ RGCs that innervate these areas undergo apoptosis, or if GFP is selectively down-regulated in these cells remains unclear. Taken together, these data suggest that Isl2-GFP RGCs not only share common molecular and morphological properties, but also selectively innervate the major image-forming, topographically-organized retinorecipient nuclei.

### Segregation of functional inputs in Isl2-EphA3 knock-in mouse

**Figure 9:**
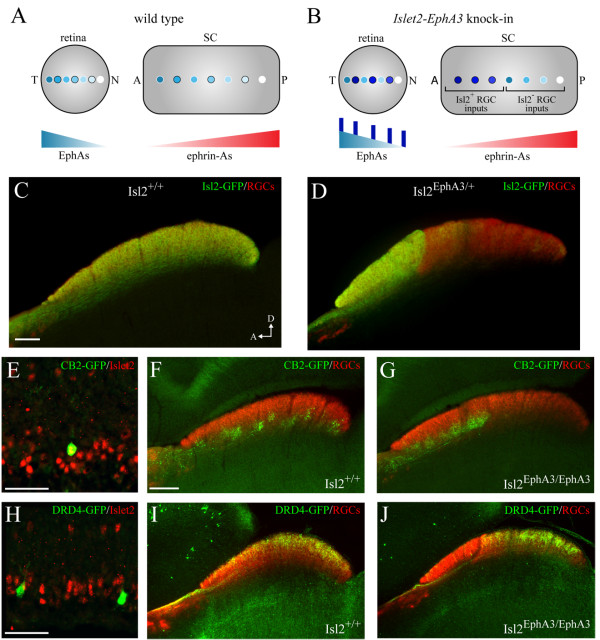
**Segregation of functionally-distinct retinal ganglion cells (RGCs) in the superior colliculus (SC) of Isl2**^**EphA3/EphA3 **^**mice. (A and B)** Schematics of topographic mapping in wild type **(A)** and Isl2^EphA3/EphA3^ mice **(B)**. In wild type mice, the temporal-nasal axis of the retina maps onto the anterior-posterior axis of the SC. In Isl2^EphA3/EphA3^, Isl2^+^ RGCs express exogenous EphA3, and thus innervate the anterior SC, while Isl2^-^ RGCs express endogenous levels of EphA receptors and innervate the posterior SC. **(C and D)** Parasagittal sections through the SC of adult (P30) wild type **(C)** and Isl2^EphA3/+^ mice **(D)** in which all RGC terminations are labeled by CTB-555 (red) and Isl2-GFP RGC terminations are labeled by immunofluorescence of GFP (green). **(E)** Immunofluorescence of Isl2 (red) expression reveals co-localization in CB2-GFP RGCs (green) in a P8 retina. **(F and G)** Parasagittal sections through the SC of adult (P30) wild type **(F)** and Isl2^EphA3/EphA3^ mice **(G)** reveal the terminations of CB2-GFP RGCs (green) in relation to all RGCs (red). **(H)** Immunofluorescence of Isl2 (red) expression reveals an absence of co-localization in DRD4-GFP RGCs (green) in a P8 retina. **(I and J)** Parasagittal sections through the SC of adult (P30) wild type **(I)** and Isl2^EphA3/EphA3^ mice **(J)** reveal the terminations of DRD4-GFP RGCs (green) in relation to all RGCs (red).

Based on the common properties of Isl2-GFP RGCs, we hypothesized that expression of Isl2 would be associated with distinct functionally-defined cell types. To test this, we determined the expression of Isl2 in two different previously-described GFP BAC transgenic lines: the CB2-GFP line that expresses GFP in a population of transient OFF-alpha RGCs, has dendrites restricted to the S3 lamina of the IPL and sends axons to a deep layer of the SC [30], and the DRD4-GFP line, which expresses GFP in a class of direction-selective RGCs that prefer movement in the posterior direction, have bistratified dendrites in the S2 and S4 laminae of the IPL and send axons to the upper layer of the SC [31]. Consistent with our characterization of Isl2-GFP RGCs, 100% (18/18) of CB2-GFP RGCs express Isl2, while only 4.1% (1/24) of DRD4-GFP RGCs express Isl2 (Figure 9D and G).

Based on the differential expression of Isl2 in CB2-GFP and DRD4-GFP RGCs, we predicted that each of these lines would project their axons to distinct domains in the *Isl2-EphA3* knock-in mouse (Isl2^EphA3/EphA3^) [23]. These mice express exogenous EphA3 in Isl2^+^ RGCs in addition to endogenous EphAs. Because RGCs sort topographically along the anterior-posterior axis of the SC based on relative EphA levels [23,32], the Isl2^+^ RGCs expressing EphA3 terminate in the anterior SC, while those expressing endogenous levels of EphA terminate in the posterior SC, creating two maps of space [33] (Figure 9A and B). Indeed, Isl2-GFP^+^ RGC terminals, which are found throughout the SC in wild type mice, are enriched in the anterior SC in Isl2^EphA3/+^ mice, consistent with both axon tracing studies and mathematical models (Figure 9C and D) [23,32,34]. To determine if RGCs of distinct functional types segregate into the different maps of space in the SC of the EphA3^ki/ki^ mouse, we crossed the CB2-GFP and DRD4-GFP lines into the Isl2^EphA3/EphA3^ background and examined their projection patterns in the SC. In wild type mice, the axons of both CB2-GFP and DRD4-GFP RGCs are found throughout the anterior-posterior axis of the SC (Figure 9F and I). In striking contrast, in Isl2^EphA3/EphA3^ mice, CB2-GFP RGC projections were restricted to the anterior (or Isl2^+^) half of the SC, while DRD4-GFP projections terminated primarily in the posterior (or Isl2^-^) half of the SC (Figure 9G and J). Together with our previous finding that the anterior and posterior domains of the SC had differential response properties to a common visual stimulus [33], these data suggest that Isl2^+^ RGCs represent a subset of RGCs sharing molecular, morphological and functional properties.

## Discussion

There are numerous types of RGCs that can be classified based on morphological, physiological, and molecular differences. The transcriptional programs by which the diversity of RGC subtypes is generated remain unknown, as do the mechanisms by which RGCs of a given class achieve precise connectivity within the retina and within their targets in the brain. In this study, we show that Isl2-expressing RGCs are a class of primarily non-ON-OFF DSGCs that are involved in image-forming circuits. Using a transgenic mouse line that expresses GFP in the cell soma, dendrites and axons of nearly all endogenously Isl2-expressing RGCs, we show that Isl2 expression is correlated with RGCs that have dendritic lamination patterns restricted to the S3 sublamina of the IPL, project axons to image-forming retinorecipient areas, and express molecular markers of alpha-RGCs, but not ON-OFF DSGCs. We also find that Isl2 is expressed in subsets of retinal interneurons that similarly show common molecular and morphological properties.

### Isl2^+^ RGCs are a related subset that participate in the major visual circuits

We used a number of criteria to conclude that Isl2^+^ RGCs represent a related subset. First, Isl2-GFP^+^ RGCs have a unique dendritic stratification pattern, in which most are monostratified in layer S3 of the IPL, and are excluded from layers S2 and S4, the location of ON-OFF DSGC dendrites. Since dendritic positioning in the IPL is a reliable indicator of the functional output of RGCs, these data strongly suggests that Isl2-GFP^+^ RGCs are functionally related. Second, we were also able to show that endogenously Isl2^+^ RGCs also stratify their dendrites in a similar pattern, enriched in the S3 lamina of the IPL. When single Isl2^+^ RGCs were filled, we found that most had dendrites that were monostratified in layer S3, although a small percentage had dendrites that were monostratified in layer S5 or S1 or bistratified in layers S3/S5. Third, we found that a vast majority (approximately 83%) of alpha-RGCs are Isl2^+^, including CB2-GFP^+^ RGCs.

In comparison to previously described, genetically-marked RGCs, Isl2^+^ RGCs morphologically resemble CB2-GFP RGCs and the so-called W3 cells. CB2-GFP RGCs are transient OFF-alpha-RGCs that have dendrites stratified in layer S3 of the IPL and project exclusively to the dLGN and SC, where they terminate in a deeper sublamina [30]. W3 RGCs are the most numerous RGC type, representing approximately 13% of all RGCs, and play a critical role in feature detection [35]. These cells are nonlinear ON-OFF RGCs that have dendrites stratified in layer S3 and project exclusively to the dLGN and SC, where they terminate in the superficial-most sublamina. Since Isl2-GFP RGCs represent approximately 40% of all RGCs, it is reasonable that this largest subtype is represented within the Isl2^+^ class.

We also examined the projection patterns of Isl2-GFP^+^ RGCs and found that they initially project to multiple retinorecipient areas before refining to terminate primarily in the SC and dLGN. The fact that Isl2-GFP^+^ RGCs selectively innervate image-forming centers and not the SCN, MTN, OPN or pretectum, suggests that these neurons do not participate in non-image forming visual circuits. However, we did find a few Isl2-GFP^+^ RGCs that terminate in the ventral LGN, a structure that mediates luminance detection and circadian rhythm entrainment [36]. Consistent with this, we found very few Isl2-GFP^+^ RGCs stained positively for the ipRGC marker, melanopsin. Based on the high proportion of Isl2-GFP^+^ RGCs that are alpha cells, we hypothesize these ipRGCs may be the M4 class, which have alpha-like characteristics [25,26]. Taken together, these morphological and molecular data suggest that Isl2 is expressed in related subsets of RGCs and that Isl2 may play an important role in either the promotion of an alpha-RGC fate or in the suppression of an ON-OFF DSGC fate.

The mechanisms by which RGC types become specified remain unclear, but our data support a model in which RGC type is established based on the expression of a combination of transcription factors. Elegant work from several labs have shown that the expression of LIM-homeodomain transcription factors play a critical role in the specification of spinal motor neurons [9]. Indeed, Isl2 itself has been implicated in visceral versus somatic motor neuron fate specification through its modulation of overall Isl transcription factor activity [15]. Here, we show that a significant number of Isl2-GFP^+^ RGCs express Brn3a, a POU family transcription factor. Brn3a^+^ RGCs project to contralateral domains of the SC and dLGN, in a strikingly similar pattern to Isl2-GFP RGCs [22]. However, Brn3a^+^ RGCs stratify dendrites in multiple laminae of the IPL (S1-S4) [11], while Isl2-GFP RGC dendrites are primarily restricted to lamina S3. Together, these findings suggest that Isl2 expression in Brn3a^+^ RGCs serves to restrict RGC lamination in the IPL. This could occur through the direct transcriptional activation of cell adhesion molecule genes that direct dendrites to lamina S3 or repression of molecules required to target other laminae. Whether Isl2 and Brn3a share common target genes is unknown and future studies investigating this will lead to insights into the mechanism by which fate specification is established.

#### *Segregation of projections of functionally-distinct RGCs in the SC of* Isl2^EphA3/EphA3^*mice*

Our previous investigations of the Isl2^EphA3/EphA3^ mice revealed that two representations of the visual world were established in the SC of these mice [33]. While each of these occupied approximately the same amount of territory, the response properties to a drifting bar stimulus were significantly stronger in the Isl2^+^ RGC-recipient map. We hypothesized that this arose from differential functional properties of the Isl2^+^ and Isl2^-^ RGCs innervating each map. To test this, we crossed the Isl2^EphA3/EphA3^ mice with lines in which GFP is expressed in either transient OFF-alpha cells (CB2-GFP) or a type of ON-OFF DSGC (DRD4-GFP). In these double transgenic mice, CB2-GFP projections are restricted to the anterior SC, whereas DRD4-GFP projections terminate in the posterior SC. This striking result confirms that Isl2^+^ and Isl2^-^ RGC populations are completely segregated in the Isl2^EphA3/EphA3^ mouse, providing further support for the roles of relative EphA signaling and competition in topographic map formation [32,37]. Interestingly, the laminar targeting of axon terminals of CB2-GFP and DRD4-GFP RGCs are unchanged in Isl2^EphA3/EphA3^ mice, suggesting that lamination and topographic refinement are separable events.

The segregation of these different pathways of visual information provides a unique tool for dissecting the structure and function of distinct retinocollicular visual circuits. Receptive fields of collicular neurons in the mouse are significantly different from those found in the retina [12], and the mechanisms by which these receptive field properties arise remain unclear. For instance, orientation-selective receptive fields in the SC could arise through targeted innervation by linear arrays of ON-center RGCs, as suggested by Hubel and Wiesel, in the primary visual cortex [38]. Alternatively, converging innervation by DSGCs preferring opposing directions could also confer orientation-selectivity, as has recently been suggested in subcortical circuits [13,39]. In the future, physiological recording techniques that measure the preferred stimulus of SC neurons in the anterior and posterior maps of Isl2^EphA3/EphA3^ mice will help us understand how different RGC types contribute to the receptive field properties of SC neurons.

### Isl2-GFP is expressed in retinal cell types other than RGCs

In addition to being expressed in RGCs, Isl2 is also expressed in distinct sets of amacrine and bipolar cells. Similar to Isl2^+^ RGCs, Isl2^+^ amacrine cell processes are primarily found in layer S3 of the IPL and all avoid layers S2 and S4. Interestingly, there are no GFP^+^ amacrine cells in the INL, suggesting that displaced and INL-residing amacrine cells are molecularly distinct. Isl2^+^ amacrine cells do not express ChAT and stratify in different IPL sublaminae than starburst amacrine cells. An intriguing possibility raised by this finding is that Isl2^+^ RGCs and Isl2^+^ amacrine cells participate in the same circuit and synapse with one another. While determining this is beyond the scope of this study, recent studies in the cortex have shown that clonally-related cells do preferentially synapse with one another [40].

While endogenous Isl2 is expressed exclusively in the GCL, we found that bipolar cells in the INL are also labeled in Isl2-GFP mice. This suggests that GFP expression derived from this BAC transgene does not perfectly match that of the endogenous gene. Interestingly, the bipolar cells labeled were all of the ON-type, expressing both ON-cone and rod bipolar markers. These bipolars terminate in the inner half of the IPL, somewhat overlapping Isl2^+^ dendrites, raising the possibility that Isl2-GFP^+^ bipolars may also participate in the same circuits as Isl2-GFP RGCs and amacrine cells.

## Conclusions

Here, we describe a novel transgenic line in which GFP is expressed in the cell soma, dendrites and axons of distinct subsets of retinal cells. We find that Isl2-GFP expression largely overlaps with that of endogenous Isl2. These molecularly labeled cells will allow for future studies of the developmental mechanisms by which dendritic and axonal guidance decisions of this subset of retina cells are mediated. Further, GFP expression is maintained well into adulthood, thus allowing for *in vitro* experiments to determine if GFP^+^ cells preferentially form synapses with one another and whether Isl2 guides circuit formation. This transgenic line will prove to be a valuable tool in future studies of the wiring mechanisms in the retina and its targets in the brain.

## Methods

### Mice

*Islet2-EphA3* knock-in mice (Isl2^EphA3/EphA3^), *Islet2-LacZ* knock-in, CB2-GFP and DRD4-GFP mice were genotyped as described previously [15,23,30,31]. Cryopreserved sperm from Isl2-GFP transgenic mice (Stock Tg(Isl2-EGFP)LW124Gsat/Mmucd) was obtained from the Mutant Mouse Regional Resource Center (MMRRC), an NIH funded strain repository, and was donated to the MMRRC by the NINDS-funded GENSAT BAC transgenic project. *In vitro* fertilization was performed at University of California, Santa Cruz (UCSC). Positive transgenic mice were determined by PCR of tail DNA using primers against GFP (5′-CCTACGGCGTGCAGTGCTTCAGC-3′ and 5′-CGGCGAGCTGCACGCTGCGTCCTC- 3′). The study was approved by and performed in accordance with the Institutional Animal Care and Use Committees at UCSC and Children’s National Medical Center.

### Immunohistochemistry

Postnatal mice were sacrificed and intracardially perfused with ice-cold PBS (in mM: 136.9 NaCl, 2.7 KCl, 10.1 Na_2_HPO_4_, 1.4 K_2_PO_4_) followed by ice-cold paraformaldehyde (PFA) (pH 7.4, 4% in PBS). Eyes were dissected out and fixed in 4% PFA for either 30 minutes at room temperature or overnight at 4°C. The eyes were washed briefly in PBS and the retinas prepared for immunostaining. For whole mount preparation, the retina was dissected out of the eye and placed in blocking buffer (10% serum, 0.25% Triton X-100 in PBS). For cryosectioning of the retina, the lens and vasculature were removed with fine forceps and the retina was sunk in 30% sucrose overnight at 4°C. The following day, retinas were embedded in Tissue-Tek OCT Compound (Sakura Finetek, USA, Torrance, CA, USA) on dry ice and stored at -80°C until sectioned. Thin sections were cut at 16 to 20 μm on a CM1520 Cryostat (Leica Microsystems, Buffalo Grove, IL, USA) maintained at -20 to -25°C and collected on histology-grade glass slides. Slides were allowed to dry overnight at room temperature and immediately used for immunostaining or frozen at -80°C until used.

For whole mount staining, retinas were incubated in blocking buffer for one hour at room temperature. The following antibodies were diluted as indicated in blocking buffer and incubated overnight at 4°C with rocking: GFP rabbit polyclonal, 1:1,000 (Life Technologies, Carlsbad, CA, USA); GFP chicken polyclonal, 1:1,000 (Aves Lab, Tigard, OR, USA); calretinin goat polyclonal, 1:500 (EMD Millipore, Billerica, MA, USA); Isl2 guinea pig polyclonal, 1:100 (Abcam, Cambridge, MA, USA); VA-ChAT goat polyclonal, 1:500 (Promega, Madison, WI, USA); ChAT goat polyclonal, 1:500 (EMD Millipore, Billerica, MA, USA); Brn3a goat polyclonal, 1:500 (Santa Cruz Biotechnology, Santa Cruz, CA, USA); Pax6 rabbit polyclonal, 1:500 (Covance, Princeton, NJ, USA); SMI-32 mouse monoclonal, 1:1,000 (Covance, Princeton, NJ, USA); melanopsin rabbit polyclonal, 1:500 (Advanced Targeting Systems, San Diego, CA, USA); PKCα rabbit polyclonal, 1:5,000 (Sigma-Aldrich, St. Louis, MO, USA); G_o_-α rabbit polyclonal, 1:1,000 (EMD Millipore, Billerica, MA, USA); Bhlhb5 goat polyclonal, 1:300 (Santa Cruz Biotechnology, Santa Cruz, CA, USA). The following day, retinas or sections were washed in PBS at room temp (5 × 1 hour for retinas, 3 × 30 minutes for sections) and incubated with appropriate fluorescently-conjugated secondary antibodies diluted at 1:1,000 in blocking buffer for 1 hour at room temperature. Retinas and sections were washed again and a coverslip was mounted using Fluoromount G (Southern Biotechnology, Birmingham, AL, USA). Imaging was performed with an Olympus BX51 epifluorescent microscope (Olympus, Center Valley, PA, USA) equipped with QImaging Retiga EXi digital camera (QImaging, Surrey, BC, Canada). Confocal images were collected on an Olympus 1X81 inverted microscope (Olympus, Center Valley, PA, USA) equipped with a Fluoview FV1000 imaging system (Olympus, Center Valley, USA). Quantification of fluorescence intensity across the IPL was achieved using the plot profile function in Image J (National Institutes of Health, Bethesda, MD). A line spanning the IPL was drawn perpendicular to the ChAT bands and centered between them. The average of three such lines in a representative sample was used to generate the plot in Figure 3E.

### Targeted cell filling and two-photon imaging

Retinas mounted on filter papers were superfused with warmed (32°C) and oxygenated artificial cerebrospinal fluid (in mM: 119 NaCl, 2.5 KCl, 1.3 MgCl_2_, 1.0 K_2_HPO_4_, 2.5 CaCl_2_, 26.2 NaHCO_3_, and 11 D-glucose). Glass microelectrode (3 to 5 MΩ) filled with an internal solution (containing 98.3 mM potassium-gluconate, 1.7 mM KCl, 0.6 mM EGTA, 5 mM MgCl_2_, 2 mM Na_2_-ATP, 0.3 mM GTP, and 40 mM HEPES, pH 7.25 with KOH, 20 μM Alexa Fluor 488 (Life Technologies, Carlsbad, CA, USA), and 3 mg/ml biocytin (Sigma-Aldrich, St. Louis, MO, USA)) was used to deliver biocytin into GFP-positive or non-GFP cells in the whole-cell patch-clamp configuration for 10 to 20 minutes. The electrodes were then carefully withdrawn, the retina fixed with 4% PFA for 15 minutes and then processed for visualization of biocytin and ChAT. The fixed retinas were washed three times in 0.01 M PBS, and were then incubated in blocking solution (1% bovine serum albumin + 0.2% Triton-X in 0.01 M PBS) for one hour at room temperature. Goat anti-ChAT antibody (Life Technologies, Carlsbad, CA, USA) was diluted 1:200 in blocking solution and added to the retina for incubation overnight at 37°C. The retinas were then washed three times in blocking solution, for 20 minutes each, and incubated in secondary antibodies: donkey anti-goat IgG-Alexa Fluor 488 (1:500) and Alexa Fluor 594 conjugated streptavidin (1:1,000) diluted in blocking solution for two hours at 37°C. Afterwards retinas were washed in blocking solution three times for 20 minutes each, rinsed with 0.01 M PBS, and then mounted onto glass slides with Vectashield (Vector, Burlingame, CA, USA). Three-dimensional image stacks containing 80 to 110 optical sections at the z-axis were collected using a two photon microscope and Fluoview software (Olympus, Center Valley, PA, USA) at 810 nm to visual Alexa 488 and Alexa 594 (GFP is not efficiently excited at 810 nm, and therefore not visible in the images) . Each optical section was resampled three times with 1 μm between sections. Images were analyzed using ImageJ (National Institutes of Health, Bethesda, MD, USA) and Metamorph (Molecular Devices, Sunnyvale, CA, USA).

### Axon tracing

To retrogradely label RGCs, P8 pups were anesthetized on ice and a small incision was made in the scalp. Two to three holes were made in the skull over the SC using a 26.5 gauge needle. Approximately 1 μL of fluorescently-conjugated cholera toxin subunit B (CTB-555, Life Technologies, Carlsbad, CA, USA) (10 mg/mL in PBS) was injected using a pulled glass pipet and Picospritzer III (Parker Instruments, Carlsbad, CA, USA) set at low pressure (approximately 5 psi) and long pulse duration (approximately 300 ms). The scalp was sealed with superglue and pups were allowed to recover in a warm incubator before being returned to their mother. After two to three days, pups were sacrificed and intracardially perfused with PBS and PFA. Eyes were dissected out and prepared for imaging as described above.

To label all retinofugal axons, juvenile (P4 to 10) or adult mice were anesthetized on ice or by subcutaneous injection of ketamine/xylazine solution (100/10 mg/kg), respectively. Approximately 1 μL of CTB-555 (2 mg/mL) was injected using a pulled glass pipet and Picospritzer III set at high pressure (approximately 30 psi) and short pulse duration (approximately 15 ms). After two to three days, mice were sacrificed and intracardially perfused with PBS and PFA. Brains were dissected out and fixed overnight in 4% PFA at 4°C. The following day, brains were briefly washed with PBS and cryopreserved in 30% sucrose at 4°C overnight. Coronal sections were cut at 100 μm with an HM430 sliding microtome (Thermo-Fisher, Waltham, MA, USA) and collected in PBS. Immunostaining for GFP was performed as described above for whole mount retinas.

## Abbreviations

ChAT: choline acetyltransferase; CTB-555: Alexafluor 555-conjugated cholera toxin subunit B; dLGN: dorsal lateral geniculate nucleus; DSCGs: direction selective retinal ganglion cells; GCL: ganglion cell layer; INL: inner nuclear layer; IPL: inner plexiform layer; ipRGCs: intrinsically-photosensitive retinal ganglion cells; Isl2: Islet2; MMRRC: Mutant Mouse Regional Resource Center; MTN: medial tegmental nucleus; OPN: olivary pretectal nucleus; PFA: paraformaldehyde; PKCα: protein kinase C-alpha; PTC: pretectal complex; RGC: retinal ganglion cell; SC: superior colliculus; SCN: suprachiasmatic nucleus; UCSC: University of California, Santa Cruz; VA-ChAT: vesicle-associated choline acetyltransferase; vLGN: ventral lateral geniculate nucleus.

## Competing interests

The authors declare that they have no competing interests.

## Authors’ contributions

JWT conceived of the study, participated in its design and coordination, carried out the axon tracing studies, participated in the immunohistochemistry, and drafted the manuscript. WW carried out the targeted cell filling and two-photon imaging. CG carried out the bulk of the immunohistochemistry. NTS participated in the immunohistochemistry. ADH provided the CB2-GFP and DRD4-GFP lines prior to their publication and helped edit the manuscript. MBF helped draft the manuscript and participated in the study’s design and coordination. DAF participated in the design and coordination of the study and helped draft the manuscript. All authors read and approved the final manuscript.
